# Implantation accuracy of novel polyimide stereotactic electroencephalographic depth electrodes—a human cadaveric study

**DOI:** 10.3389/fmedt.2024.1320762

**Published:** 2024-02-22

**Authors:** Aura Kullmann, Farida Akberali, Jaime J. Van Gompel, Robert A. McGovern, W. Richard Marsh, Debra Kridner, Camilo A. Diaz-Botia, Michael C. Park

**Affiliations:** ^1^ NeuroOne Medical Technologies, Eden Prairie, MN, United States; ^2^ Zimmer Biomet, Warsaw, IN, United States; ^3^ Department of Neurosurgery, Mayo Clinic, Rochester, MN, United States; ^4^ Department of Neurosurgery, University of Minnesota Medical Center, Minneapolis, MN, United States; ^5^ Department of Neurology, University of Minnesota Medical Center, Minneapolis, MN, United States

**Keywords:** usability, sEEG, implantation accuracy, epilepsy, stereotactic implantation, ex-vivo models, sEEG-guided radiofrequency ablation

## Abstract

**Introduction:**

Stereoelectroencephalography (sEEG) is a minimally invasive procedure that uses depth electrodes stereotactically implanted into brain structures to map the origin and propagation of seizures in epileptic patients. Implantation accuracy of sEEG electrodes plays a critical role in the safety and efficacy of the procedure. This study used human cadaver heads, simulating clinical practice, to evaluate (1) neurosurgeon's ability to implant a new thin-film polyimide sEEG electrode according to the instructions for use (IFU), and (2) implantation accuracy.

**Methods:**

Four neurosurgeons (users) implanted 24 sEEG electrodes into two cadaver heads with the aid of the ROSA robotic system. Usability was evaluated using a questionnaire that assessed completion of all procedure steps per IFU and user errors. For implantation accuracy evaluation, planned electrode trajectories were compared with post-implantation trajectories after fusion of pre- and postoperative computer tomography (CT) images. Implantation accuracy was quantified using the Euclidean distance for entry point error (EPE) and target point error (TPE).

**Results:**

All sEEG electrodes were successfully placed following the IFU without user errors, and post-implant survey of users showed favorable handling characteristics. The EPE was 1.28 ± 0.86 mm and TPE was 1.61 ± 0.89 mm. Long trajectories (>50 mm) had significantly larger EPEs and TPEs than short trajectories (<50 mm), and no differences were found between orthogonal and oblique trajectories. Accuracies were similar or superior to those reported in the literature when using similar experimental conditions, and in the same range as those reported in patients.

**Discussion:**

The results demonstrate that newly developed polyimide sEEG electrodes can be implanted as accurately as similar devices in the marker without user errors when following the IFU in a simulated clinical environment. The human cadaver ex-vivo test system provided a realistic test system, owing to the size, anatomy and similarity of tissue composition to that of the live human brain.

## Introduction

Drug refractory epilepsy (DRE) impacts approximately 30% of more than 50 million epilepsy patients worldwide (1). For these patients who suffer from focal seizures that cannot be controlled by medication, surgery to remove the epileptogenic zone (EZ) is a main option. Stereoelectroencephalography (sEEG) electrodes are routinely implanted in the brain as part of the pre-surgical evaluation workup, to precisely identify the EZ and define the extent of the epileptic region (2).

There are several sEEG electrodes commercially available in the USA (and other countries). They are mostly silicone-based and the fabrication process involves manual steps. This results in inconsistent product and prolonged fabrication times. Here we describe a new sEEG electrode manufactured using a relatively new thin-film technology, which allows automated fabrication of electrodes. The automation minimizes inconsistencies, ensures uniformity of the product, reduces costs and increases production volume. The sEEG electrode described here has one of the smallest diameters in the market, 0.8 mm. By comparison, other sEEG electrodes range from 0.8–1.5 mm in diameter. In addition, this electrode has recently been cleared by the Food and Drug Administration (FDA) to perform sEEG-guided radiofrequency ablation (RFA) when coupled to an RF generator and a temperature accessory probe (K231675). This is the only sEEG-guided RFA system capable of monitoring the temperature during ablation. As the sEEG-guided RFA procedure is typically performed after the sEEG monitoring phase is completed, using the same already implanted sEEG electrodes, implantation accuracy of these electrodes is critical to ensure successful clinical outcomes. Any newly developed sEEG electrode must undergo extensive testing prior to obtaining FDA clearance for use in human. This testing includes biocompatibility, mechanical performance, electrical safety, sterilization, as well as usability and implantation accuracy, which are described in this study. Usability assesses whether the intended users (neurosurgeons), can operate the device, i.e., perform the tasks necessary for the sEEG electrode implantation and removal in a safe, effective and efficient manner. This testing helps identify and correct errors that can directly or indirectly harm patients or users.

The accuracy of sEEG electrode implantation is a critical factor for the precise localization of the EZ. Trajectory planning is individualized to each patient and designed to ensure electrode placement in/nearby the potential EZ(s), maximize the area of gray matter sampled which is thought to be responsible for seizure generation, and avoid vasculature (3). Inaccurate electrode implantation may result in failure to identify the EZ, insufficient data collection which can delay or impact clinical decisions, improper treatment areas if sEEG-guided RFA is performed, and complications such as intracranial hemorrhage (4). A number of factors have been shown to play a role in the implantation accuracy [for a review see Philipp LR et al., 2021 (5)]. These include errors involving the implantation systems (e.g., frameless, frame-based or robot-assisted), neuronavigational system (e.g., misregistration between planning and registration scans), trajectory angle and length (e.g., shallow entry angles increase the chances of the drill bit slipping at the start of drilling), electrode-tissue interference (e.g., electrode deviations due to structural and biomechanical properties of soft tissue such as heterogeneity, angle when crossing tissue interfaces), surgical technique (e.g., electrode deviations affected by surgical technique such as the use of stylet or anchor bolt), electrode properties (e.g., deviations affected by mechanical properties of the electrodes), and post-implantation physiological response (e.g., cerebrospinal fluid leak, tissue swelling) (4–11). Two meta-analysis studies have showed that the robot—assisted sEEG electrode implants tend to have better accuracy than frameless or frame-based systems (4, 9). A study investigating the angle of the planned trajectory has found that trajectories with a planned angle of >30 had significantly higher EPEs and TPEs than trajectories with planned angles <30 (6, 11). Regarding the surgical technique for opening the pathway through the brain parenchyma, there are two main types of sEEG electrodes, with internal stylet (e.g., Ad-Tech, Integra LifeSciences and the present electrode) and without internal stylet (e.g., PMT and Dixi Medical). A study directly comparing the implantation accuracy of internal vs. external stylet technique showed some differences in that the internal stylet technique exhibited a larger target radial error and angular deviation with a smaller depth error than the external stylet technique (10).

Both implantation accuracy and usability testing are critically dependent on using the appropriate test model and environment to mimic clinical practice. The test model should mimic as closely as possible the human brain anatomy in size, and tissue structure and properties. Also, the test environment should mimic as closely as possible the actual implantation procedure of the sEEG electrodes in the operating room using the current implantation methods, workflow and stereotactic procedures.

Given the importance of implantation accuracy and usability in clinical outcomes, the goal of this study was to evaluate these factors for a new thin-film polyimide sEEG electrode, implanted according to the instruction for use (IFU) in a fresh cadaver head with the aid of stereotactic robot-assisted implantation equipment.

## Material and methods

### Facilities

This study was performed at the American Preclinical Services (APS, Minneapolis, MN, now part of NAMSA) with the approval of the APS ethical committee and following the US FDA guidance for usability studies (FDA 2016). APS is AAALAC and ISO-17025 accredited, USDA registered and GLP-compliant Contract Research Organization.

### Materials

**Figure 1:**
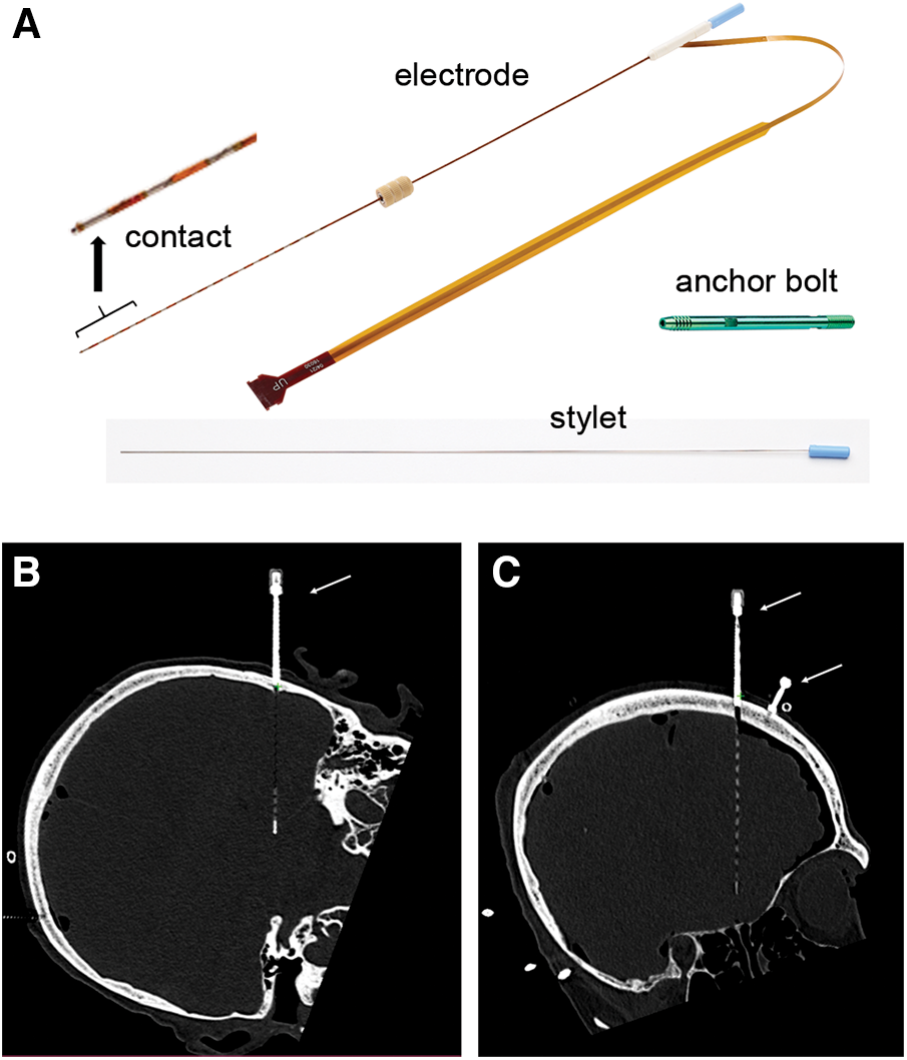
sEEG electrode and anchor bolt. (**A**) Picture of an sEEG electrode, stylet and anchor bolt. The electrode shown in this picture has 16 contacts. The anchor bolt shown here is 40 mm long. Inset shows high magnification of the contacts. (**B,C**) sEEG electrode and anchor bolt are readily visible in CT scan images. (**B**) shows an example of a 5-contact electrode and (**C**) shows an example of a 16-contact electrode. Note the visibility of the electrode tip and individual contacts. Anchor bolts are indicated by arrows.

sEEG electrodes consisting of platinum contacts on polyimide substrates were manufactured by NeuroOne Medical Technologies Corporation (Eden Prairie, MN) (Figure 1). This study used two electrode models. Model one was 80 mm in recording length with 16 contacts, contact height of 2 mm and contact spacing of 3.2 mm (*n* = 12 electrodes). Model two was 16 mm in recording length with 5 contacts, contact height of 2 mm and contact spacing of 1.5 mm (*n* = 12 electrodes). The sEEG electrodes are 0.8 mm in diameter and have an internal stylet to provide sufficient rigidity during insertion into the brain. The stylet was removed after the insertion. Anchor bolts (40 mm length, 2.4 mm outer diameter, tapered at the end that is inserted into the bone to prevent plunging into the brain, made of titanium; NeuroOne Technologies Corporation) (Figure 1) were used to guide the placement and stabilize/secure the electrodes in the brain.

### Test system and experimental design

Two fresh adult human cadavers (one male, one female) were obtained by the APS from the Anatomy Bequest Program at the University of Minnesota, a voluntary body donation program. Study conduct, data acquisition and analysis followed the APS ethical committee approved protocol. The cadaver heads were implanted with 24 electrodes, 12 electrodes per head. Four neurosurgeons (JJVG, RM, WRM, MCP) with expertise in sEEG surgical technique, each implanted 6 electrodes into one hemisphere.

### Trajectories

Trajectory planning was performed by the neurosurgeons based on their clinical practice. The trajectories were designed to reach common anatomical locations such as the amygdala, anterior and posterior hippocampus, orbitofrontal cortex, cingulate gyrus, and anterior and posterior insula. Because the implantation accuracy is influenced by the length and angle of the trajectory, the implantation plan was designed to incorporate approximately 50% long (>50 mm) and 50% short (<50 mm) trajectories and within each length a mixture of orthogonal and oblique angles of approach, while targeting the above named anatomical locations. The implanted trajectories consisted of 11 short and 13 long trajectories, with 12 orthogonal (6 short and 6 long) and 12 oblique (5 short and 7 long) angles of approach (Table 1). Orthogonal trajectories were defined as perpendicular to the midsagittal plane defined by the anterior and posterior commissure line. The remainder of the trajectories were considered oblique. The angle of the oblique trajectories was not measured. Trajectory planning was performed using ROSA Brain software (version 3.1.4.1650, Zimmer Biomed) using Computed Tomography (CT, Siemens Somatom® Definition) acquired images.

**Table 1:** Trajectory type and length.

Trajectory type	*N*	Average length (mm)	Range (mm)
		(mean ± SD)	
Short orthogonal	6	40.8 ± 5.2	33.8–48.2
Short oblique	5	42.0 ± 5.0	33.8–46.8
Long orthogonal	6	69.9 ± 15.3	57.5–91.0
Long oblique	7	66.2 ± 14.0	51.0–86.0

### Surgical placement technique

**Figure 2:**
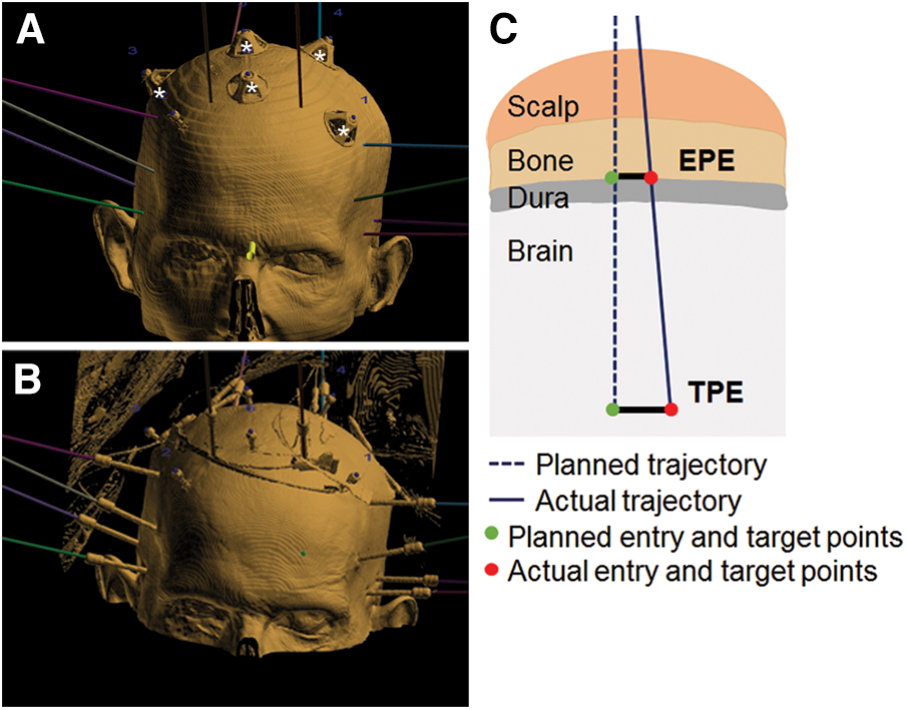
Fiducials, implant and error measurements. (**A**) 3D view illustrating fiducials (marked by white stars) and planned trajectories (colored lines). (**B**) Example of electrode positioning. Fusion of CT scan images and ROSA trajectory planning software. (**C**) Schematic of EPE and TPE measurements.

Prior to electrode placement, 5 to 6 skull-based fiducials (Medtronic Unibody Bone Fiducials 10 mm and 13 mm, Medtronic, NM) were placed in different areas of the scalp (Figures 2A,B). A CT scan was performed and the images were used for trajectory planning. The head was stabilized using a Mayfield head clamp. Stereotactic equipment (ROSA robot instrument guide; Zimmer Biomed) was prepared and used to aid in accurate device placement. The anchor bolt placement location was marked through the stereotactic ROSA guiding system according to the trajectory planning. The skin was opened with a small incision and a burr hole was drilled into the skull using a 2.1 mm drill bit. The drill bit was removed, and the anchor bolt was inserted into the skull using a compatible driver. Electrode insertion depth was obtained from the ROSA system and the electrode was set for the desired depth. The electrode with the internal stylet in, was inserted into the brain through the anchor bolt until it reached the desired depth. The stylet was then removed leaving the electrode in place. The electrode cap was tightened onto the anchor bolt, stabilizing the electrode. The electrode tail was secured to the scalp using a 3-0 Nylon suture. A CT scan was performed to verify electrode position and the acquired images were used for implantation accuracy calculation. Both the electrode and anchor bolt were clearly visible in the CT scan images (Figures 1B,C).

### Implantation accuracy data analysis and statistics

**Figure 3:**
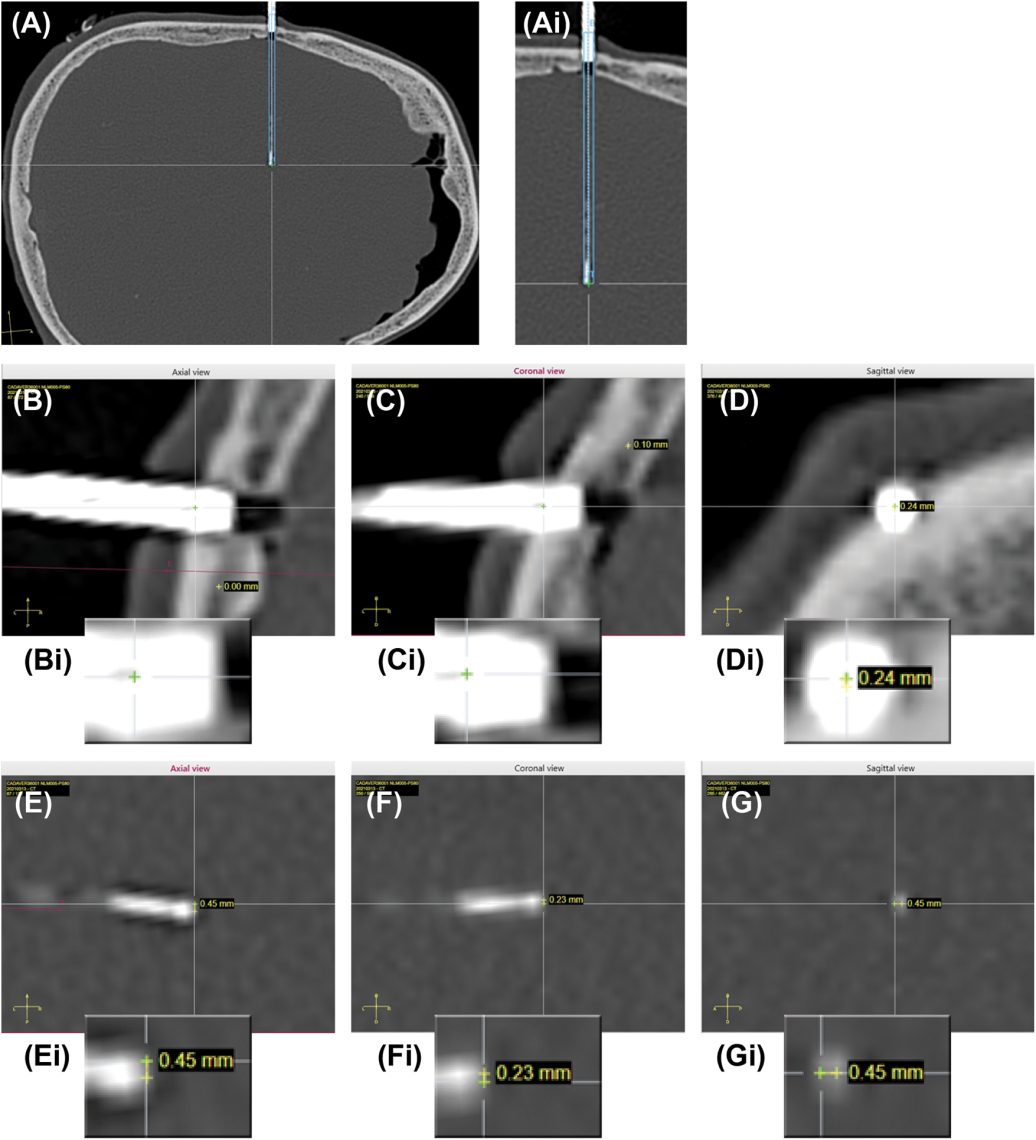
Planned and actual trajectories. (**A**) CT scan image of an actual implanted electrode is co-registered with the planned trajectory (dotted blue lines) and expected electrode location (solid blue line). Ai. Higher magnification of the actual implanted electrode and planned trajectory. (**B–D**) Example of EPE measurements in all 3 planes (axial, sagittal, coronal). Bi-Di Insets showing higher magnification images of the entry points. Yellow cross is planned trajectory, green cross is actual trajectory. The difference is the error, shown for each plane. (**E–G**) Example of TPE measurements in all 3 planes (axial, sagittal, coronal). Ei-Gi Insets showing high magnification images of the target points. Yellow cross is planned trajectory, green cross is actual trajectory. The difference is the error, shown for each plane.

Implantation accuracy was evaluated by calculating the error between the planned and final implanted location for each electrode (schematic in Figure 2C), according to the methods described in previous studies (7, 12). Entry point error (EPE) represents the difference between the actual and planned position at which the electrode passes through the skull. Target point error (TPE) represents the difference between the actual and planned position of the electrode at the target site. EPE and TPE were defined at the outer table of the bone and at the position of most distal contact, respectively, similar to previous studies (7). Pre- and post- implantation CT images were aligned, and for each electrode the EPE and TPE were manually marked using the available ROSA software measurement tools (ROSA Brain version 3.1.4.1650) (Figure 3). Differences between planned and observed entry points and targets were calculated by axis (*x*, *y*, and *z*) using ROSA software. The Euclidian distance between planned and observed points was calculated for each electrode, according to the formula:

$$D=({\left(x_{\mathrm{planned}}-x_{\mathrm{observed}}\right)}^{2}+(y_{\mathrm{planned}}-y_{\mathrm{observed}}{)}^{2}+{\left(z_{\mathrm{planned}}-z_{\mathrm{observed}}\right)}^{2}{)}^{1/2}$$

Statistical significance was tested using unpaired two tail *t*-test or nonparametric ANOVA followed by Dunn's multiple comparison tests, as described in each figure legend, using Prism-GraphPad (version 6.03 for Windows). *P* < 0.05 was considered statistically significant. Data normality was verified using Shapiro-Wilk test. Data sets that were not normally distributed were log transformed.

### sEEG electrode usability assessment

User interface with the device was assessed by evaluating the users' ability to perform the procedure according to the IFU. Four steps were evaluated: (1) preparation, which involved inspection of the electrodes and equipment; (2) anchor bolt placement, which consisted of drilling and positioning the anchor bolt into the skull; (3) sEEG electrode placement, which consisted of insertion of the electrodes into the brain according to the planned trajectories; and (4) sEEG removal, which consisted of removal of the electrodes from the brain. For each step, a usability questionnaire captured user errors, completion of the procedure as planned and any other observations. The protocol and execution followed the FDA (13) and IEC 62366-1 (14) guidance for usability.

## Results

### Trajectories

A total of 24 trajectories, 11 short (ranging from 33.8–48.2 mm) and 13 long (ranging from 51.0–91.0 mm), were inserted (Table 1). Among these, 12 trajectories were orthogonal (i.e., perpendicular to the midsagittal plane defined by the anterior and posterior commissure line) ranging from 33.8–91.0 mm and 12 oblique (i.e., not perpendicular to the midsagittal plane defined by the anterior and posterior commissure line) ranging from 33.8–86.0 mm (Table 1).

### Implantation accuracy

Implantation accuracy data are summarized in Table 2. The mean and standard deviation (SD) for the EPEs were 1.28 ± 0.86 mm, with values ranging from 0.31 mm to 2.82 mm. The mean and SD for the TPEs were 1.61 ± 0.89 mm, with values ranging from 0.31 mm to 3.92 mm. Data were summarized by the length of the trajectory (short vs. long, Table 3) and type of trajectory (orthogonal vs. oblique, Table 4). There was a significant difference between the short and long trajectories for both EPEs and TPEs (Table 3). There was no statistically significant difference between EPEs and TPEs for orthogonal and oblique trajectories (Table 4). There were no differences in accuracy between users, with the exception of EPE between user 1 and user 4.

**Table 2:** Summary of EPE and TPE.

Measure (mm)	Mean ± SD (*N* = 24)	Mean (95% CI) (*N* = 24)
EPE		
ED	1.28 ± 0.86	1.28 (0.92, 1.64)
X	0.90 ± 0.74	0.90 (0.61, 1.20)
Y	0.72 ± 0.62	0.72 (0.47, 0.97)
Z	0.17 ± 0.28	0.17 (0.92, 1.64)
TPE		
ED	1.61 ± 0.89	1.61 (1.24, 1.99)
X	0.69 ± 0.52	0.69 (0.48, 0.90)
Y	1.02 ± 0.84	1.02 (0.69, 1.36)
Z	0.70 ± 0.65	0.70 (0.43, 0.95)

**Table 3:** Comparisons between long and short trajectories.

	Short trajectories^a^	Long trajectories^b^	*p* *
Measure (ED, mm)	Mean ± SD	Mean ± SD	
	(*N* = 11)	(*N* = 13)	
EPE	0.77 ± 0.42	1.71 ± 0.90	0.0046**
TPE	1.10 ± 0.70	2.05 ± 0.79	0.0057**

^a^ Short trajectories (<50 mm): average length 41.4 ± 4.9 mm.

^b^ Long trajectories (>50 mm): average length 67.9 ± 14.1 mm.

* Statistical significance was tested using unpaired *t*-test.

** Values of *p* < 0.05 were considered significant.

**Table 4:** Comparisons between orthogonal and oblique trajectories.

	Orthogonal trajectories^a^	Oblique trajectories^b^	*p* *
Measure (ED, mm)	Mean ± SD	Mean ± SD	
	(*N* = 12)	(*N* = 12)	
EPE	1.51 ± 1.01	1.04 ± 0.61	0.1851
TPE	1.67 ± 1.02	1.56 ± 0.76	0.7685

^a^ Orthogonal trajectories were defined as perpendicular to the midsagittal plane defined by the anterior and posterior commissure line.

^b^ Oblique trajectories were defined as not perpendicular to the midsagittal plane. Orthogonal trajectories: average length 55.4 ± 18.7 mm; Oblique trajectories: average length 56.1 ± 16.5 mm. Statistical significance was tested using unpaired *t*-test.

* Values of *p* < 0.05 were considered significant.

### sEEG electrode usability assessment

All users were able to follow the instructions for use and perform the four steps of the procedure: (1) preparation, which involved inspection of the electrodes and equipment. This step confirmed that the electrodes were not damaged, were sterile and within the expiration date, and the additional tools and equipment needed for implant were compatible with the electrodes. (2) anchor bolt placement, which consisted of drilling a 2.1 mm burr hole into the bone and positioning the anchor bolt into the skull; (3) sEEG electrode placement, which consisted of inserting the electrodes into the brain according to the planned trajectories; and (4) sEEGs and anchor bolts removal, which consisted of removal of the electrodes from the brain and anchor bolts from the skull. There were no errors, no deviations and no difficulties noted (Table 5).

**Table 5:** Summary of usability assessment. User interface with the device was assessed by evaluating the users’ ability to perform the procedure according to the instructions for use (IFU). The four steps: preparation, anchor bolt placement, electrode placement and sEEG system removal, were repeated by each user for each electrode (6x/user).

User (Surgeon initials)	User 1 (MCP)	User 2 (RAM)	User 3 (JJVG)	User 4 (WRM)
Donor ID	35,996	35,996	36,001	36,001
Steps evaluated	Errors and other observations (repeated 6x for each electrode/surgeon)			
1. Preparation: inspection of the electrodes and equipment	No damage	No damage	No damage	No damage
	No error	No error	No error	No error
2. Anchor bolt placement: drilling and positioning the anchor bolt into the skull	Slight taper to anchor bolt making it difficult to tighten. Anchor bolt could be felt as securely tightened in the temporal bone.	No error	No error	No error
3. Electrode placement: insertion of the electrodes into the brain according to the planned trajectories	No error	No error	No error	No error
4. sEEG system removal: remove electrodes and anchor bolts from the brain	No error	No error	No error	No error

## Discussion

This study characterized implantation accuracy and usability (user interaction with the device) of a newly developed thin-film polyimide sEEG electrode in a simulated clinical environment. The study used a cadaver head as a test system. Users (neurosurgeons) implanted sEEG electrodes with the aid of the ROSA robotic system using current clinical implantation methods, workflow and stereotactic procedures, in a research operating room. The results demonstrated that the new electrodes can be placed accurately with excellent usability, following the IFU. The EPEs and TPEs were in the range of those reported in a similar cadaveric study (12) and in patients (4, 15–18) when using similar methodology (Tables 6A,B).

**Table 6:** Comparisons of EPEs and TPEs reported in the literature in cadaver (A) and patient (B).

A. Comparisons of EPEs and TPEs in cadaver studies				
Technique, equipment	EPE	TPE	N electrodes/trajectories	Reference
	Mean (95% CI) or as indicated	Mean (95% CI) or as indicated		
NeuroOne sEEG	1.28 (0.92, 1.64)	1.61 (1.24, 1.99)	24	Present study
Robotic (ROSA)				
Electrode A	0.91 (−1.12, 1.93)	1.45 (0.73, 2.17)	52	(12)
Robotic (ROSA)				
Electrode B	1.83 (0.80, 2.85)	4.32 (3.60, 5.05)	52	(12)
Robotic (ROSA)				
B. Comparisons of EPEs and TPEs in patient studies				
Technique, equipment	EPE	TPE	N electrodes/trajectories	Reference
	Units as indicated	Units as indicated		
Robotic (ROSA) Electrode A	mean ± SD	mean ± SD	** **	** **
	0.7 ± 0.5	1.6 ± 0.8	n/a	(19)
	1.62 ± 1.8	2.66 ± 2.3	813	(20)
Robotic (ROSA) Electrode B	n/a	n/a	n/a	n/a
Robotic (ROSA) Electrode C	mean ± SD	mean ± SD	40	(16)
	2.53 ± 0.24	2.96 ± 0.24		
	range 0.31–6.38	range 1.04–7.51		
Robotic (ROSA) Electrode D	Median (IQR):	Median (IQR):	500	(15)
	1.2 (0.78–1.83)	1.7(1.20–2.30)		
	range: 0.3–5.1	range: 0.4–7.1		
With or without robotic assistance, frame and frameless Electrodes—different types	n/a	2.33(2.087, 2.586 95% CI)	3,647	(5) Meta-analysis
		range: 1.64–4.05		
Robotic Electrodes different types	1.17 (0.80–1.53 95% CI)	1.71 (1.66–1.75 95% CI)	n/a	(4) Meta-analysis
Manual frame-based, Electrodes different types	1.43 (1.35–1.51 95% CI)	1.93 (1.05–2.81 95% CI)	n/a	(4) Meta-analysis
Frameless systems Electrodes different types	2.45 mm (0.39–4.51 95% CI)	2.89 mm (2.34–3.44 95% CI)	n/a	(4) Meta-analysis

All distances are measured in mm. IQR, interquartile range.

Electrodes: A- Dixi Medical, B- Integra LifeSciences, C- Ad-Tech Medical; D-PMT Corp.

### Implantation accuracy

Precise localization of the EZ as well as effective treatment when using sEEG-guided RFA rely on implantation accuracy of sEEG electrodes. Consequently, numerous studies have investigated the implantation accuracy of different sEEG electrodes using *in vitro* systems (e.g., phantom) (21–24), cadavers (12) and patients [for reviews see (4, 5, 9)], and have identified a number of factors that can affect it. These factors include electrode properties, trajectory length and type, surgical equipment used for implantation, registration and referencing methods, and others.

Electrode properties that influence EPEs and TPEs include the use of a stylet and use of a guiding bolt (also known as anchor bolt) for insertion (10). A previous study has investigated the implantation accuracy of two different electrodes, electrode A (from Dixi Medical) and electrode B (from Integra Lifesciences), using similar methods to those used in our study, i.e., electrodes implanted in cadavers with the aid of ROSA robotic system (12). Electrode A was placed using a guiding bolt with the aid of an external stylet. The external stylet was used to first create a pre-path for the electrode insertion, then the stylet was removed, and the electrode inserted along the created path. Electrode B was placed without a guiding bolt but had an internal stylet which ensured sufficient rigidity during insertion, with a single path. The internal stylet was then removed, leaving the electrode in place. The electrodes used in the present study are characterized by an internal stylet (like electrode B) and guiding bolts (like electrode A), and have been implanted using similar methodology and test system (i.e., ROSA system, cadaver heads). Comparisons of the data obtained in our study with data from electrodes A and B (Table 6A) show that the EPEs and TPEs of the new thin-film polyimide sEEG electrode are not significantly different from those obtained with electrode A, and TPEs were smaller than those obtained with electrode B. Thus, using similar implantation techniques, our study demonstrates comparable or higher accuracy with that of two types of commercially available sEEG electrodes. Given similar accuracy to that of the electrode A, one advantage of this new thin-film polyimide sEEG electrode is that a single insertion path is needed, as opposed to two penetrations (stylet followed by electrode). This has the potential to reduce the risk for hemorrhage and shorten the duration of the procedure. When compared to electrode B, the new thin-film polyimide sEEG electrode has a higher accuracy and a much smaller diameter (almost half of the diameter of the electrode B: 0.8 mm vs. 1.5 mm). This has the potential to reduce the risk for hemorrhage and other complications due to electrode diameter.

Clinical studies have been using electrodes similar to ours, with an internal stylet (e.g., electrode B from Integra and electrode D from Ad-Tech) as well as electrodes with an external stylet (e.g., electrode A from Dixi Biomedical, electrode C from PMT) (Table 6B). Lee at al., 2023, compared electrodes C and D and found that better target radial accuracy was achieved when using the external stylet electrodes (electrode C) (10). Other studies also reported EPE and TPE for the specific electrodes (Table 6B). For the same robot-assisted systems (ROSA), the EPE values ranged from 0.7 ± 0.5 mm (mean ± SD) (19) and 2.66 ± 2.3 (mean ± SD) (20) for electrode A, to 2.53 ± 0.24 mm (mean ± SD) (16) for electrode C, and to 1.2 mm median with 0.78–1.83 mm interquartile range (15) for electrode D. The TPE values ranged from 1.6 ± 0.8 mm (mean ± SD) (19) and 1.62 ± 1.8 (mean ± SD) (20) for electrode A, to 2.96 ± 0.24 mm (mean ± SD) (16) for electrode C, and to 1.7 mm median with 1.20–2.30 mm interquartile range (15) for electrode D. These studies do not clearly support a consistent relationship between the errors and type of electrodes, but there are many other confounding factors (e.g., anatomical locations targeted, surgeons, and others).

Trajectories length (short vs. long) and type (orthogonal vs. oblique) have been shown to play a role in accuracy. Previous studies (6) have shown that long trajectories (>50 mm) resulted in higher TPEs. Our results are similar, with both EPEs and TPEs being larger for long vs. short trajectories (Table 3). Previous studies have also shown that the angle at which the electrode is introduced can influence both EPE and TPE. For example, oblique trajectories with an insertion angles less than 30 degrees resulted in larger errors than those of the orthogonal trajectories (6, 11), especially for the long trajectories (10). In our study, there were no significant differences between oblique and orthogonal trajectories. The differences may be due to the system (cadaver vs. patient). Equipment and techniques used, e.g., frame-based, frameless or robot-assisted systems, can influence implantation accuracy. Previous studies have described sEEG implantation accuracy qualitatively and quantitatively comparing positioning technique (robot-assisted system vs. mechanical arm) and stereotactic frames vs. frameless image-guided systems [for reviews see (4, 5, 9)]. Overall, these studies suggest that robot-assisted systems provide a more accurate method of implantation, though there is variability between studies, equipment used, imaging used (CT vs. MRI) and others. For example, Cardinale et al. 2013, found significant improvement in both the entry point and target point accuracy when using the NeuroMate robotic system as compared to the Talairach frame (25). Other studies, such as Gonzalez-Martınez et al. 2016, found no significant differences for EPE when using the ROSA robotic system as compared to the Leksell frame (15). Two meta-analysis studies report that robotic assistance (ROSA, NeuroMate, others) results in better accuracy when compared to frame based stereotactic non robotic methods (4, 5) (Table 6B). For example, using robotic trajectory guidance systems EPE was 1.17 mm (0.80–1.53 95% CI) and TPE 1.71 mm (1.66–1.75 95% CI). By comparison, for the frame-based systems, mean EPE was 1.43 mm (1.35–1.51 95% CI) and mean TPE was 1.93 mm (1.05–2.81 95% CI). For the frameless systems mean EPE was 2.45 mm (0.39–4.51 95% CI), and mean TPE was 2.89 mm (2.34–3.44 95% CI) (4). All types of electrodes (e.g., Dixi Medical, Ad-Tech, Integra LifeSciences, PMT) were used in these studies, with no breakdown by electrode type. Our study used ROSA robot-assisted system and although we cannot compare the results obtained in a cadaver study with results from patients, the EPEs and TPEs are smaller or similar to those reported in the literature (Tables 2, 6A,B).

### Usability assessment

The FDA (13), IEC 62366-1 (14) and European Regulatory Agencies provide guidance and standards for testing human factors and usability engineering processes. The goal is to maximize the likelihood that new medical devices are safe and effective for the intended users, uses and use environments. Our protocol design and testing followed these requirements. We evaluated all aspects of usability testing, which included users, environments, and device user interface. The device users were neurosurgeons that routinely implant sEEG electrodes. The procedure used one of the robot-assisted systems (ROSA) that is available in many hospitals and the environment simulated an operating room. All aspects of a typical clinical workflow were followed, from inspection of the packages and equipment to fiducial placement, trajectory planning, implantation of the anchor bolts, insertion of the sEEG electrodes, and removal of the sEEG electrodes and anchor bolts. All tasks were correctly executed, indicating that the newly developed sEEG electrodes can be safely integrated in the existing clinical workflow.

### Importance of the test system

The choice of an appropriate test system is critical in the evaluation of both accuracy and usability. We used a human cadaver as the test system because it mimics closely the human brain size, anatomy and structure. *In vivo* large animal models may be informative, however, the significant difference between the size of the brain does not allow proper testing. For example, the dimensions of a pig brain are approximately 50 × 70 × 50 mm. The length of the trajectories in human routinely includes trajectories longer than 50 mm. As the implantation error is influenced by the length of the trajectory [Table 3 and (6)], brain size is critical. Phantoms have been used in previous studies (21–24), however they are frequently made of homogeneous media, which do not equate human brain consistency and structures, i.e., gray/white matter interfaces, dura, gyri, ventricles, which surgeons need to navigate when implanting sEEG electrodes. While the cadaver model lacks *in vivo* tissue properties, like blood flow and elasticity, the implantation accuracy results are in line with results obtained in patients (Tables 6A,B), suggesting that this is an appropriate model for this type of testing.

## Conclusion

This study has shown that newly developed thin-film polyimide sEEG electrodes can be implanted according to the IFU without user errors in a simulated clinical environment, i.e., a human cadaver. While many factors may affect the implantation accuracy, the results suggest that the electrodes can be implanted accurately, with accuracies similar or superior to those reported in the literature when using similar experimental conditions. The cadaver head as a testing system, is adequate for the assessment of device user interface as well as evaluation of implantation accuracy.

## Data Availability

The original contributions presented in the study are included in the article/supplementary materials, further inquiries can be directed to the corresponding author.
